# Cost-Effectiveness of One-time Universal Testing for Hepatitis D Among Adults With Chronic Hepatitis B in the United States

**DOI:** 10.1093/cid/ciaf181

**Published:** 2025-04-08

**Authors:** Mehlika Toy, David Hutton, Eyasu Teshale, William W Thompson, Hang Pham, Joshua A Salomon, Samuel So

**Affiliations:** Erasmus School of Health Policy and Management, Erasmus University, Rotterdam, The Netherlands; Department of Health Management and Policy, University of Michigan, Ann Arbor, Michigan, USA; Division of Viral Hepatitis, Centers for Disease Control and Prevention, Atlanta, Georgia, USA; Division of Viral Hepatitis, Centers for Disease Control and Prevention, Atlanta, Georgia, USA; Asian Liver Center, Department of Surgery, Stanford University School of Medicine, Stanford, California, USA; Department of Health Policy, Stanford University School of Medicine, Stanford, California, USA; Center for Health Policy, Freeman Spogli Institute for International Studies, Stanford University, Stanford, California, USA; Asian Liver Center, Department of Surgery, Stanford University School of Medicine, Stanford, California, USA

**Keywords:** delta hepatitis, HDV, screening, viral hepatitis, cost-effectiveness

## Abstract

**Background:**

Chronic hepatitis D virus (HDV) infection increases the risk of liver-related deaths in adults with chronic hepatitis B (CHB). In the United States (US), only an estimated 12.9% of adults with CHB have received an HDV antibody test. The aim of this study is to calculate the cost-effectiveness of one-time universal HDV testing of hepatitis B surface antigen (HBsAg)–positive adults living in the US.

**Methods:**

A Markov model was used to calculate the costs, health impact, and cost-effectiveness of universal testing of HBsAg-positive adults with an HDV antibody test and, when positive, an HDV RNA test for chronic HDV infection. We assumed that 50% of the HDV RNA–positive patients would receive the current recommended treatment with pegylated interferon (PEG-IFN) for 48 weeks with a 30% response rate. We also modeled the potential impact of hypothetical indefinite HDV antiviral therapy with a higher response rate to assess the annual cost threshold to be considered cost-effective.

**Results:**

Universal HDV testing of adults with CHB could avert 100 HDV-related deaths and an additional 30 cases of cirrhosis and 50 cases of hepatocellular carcinoma, and potentially result in a gain of 1500 quality-adjusted life-years (QALYs) per 100 000 HBsAg-positive individuals screened. At a willingness-to-pay threshold of $50 000 per QALY, the annual drug costs for a hypothetical indefinite therapy with a 50% or 70% treatment response rate would need to cost ≤$13 027 and ≤$14 104, respectively.

**Conclusions:**

One-time HDV testing for all HBsAg-positive adults and treatment of chronic HDV infection with PEG-IFN is potentially cost-effective in the US.

## Teaser

One-time testing of adults with chronic hepatitis B infection for hepatitis D in the United States is likely cost-effective and will result in better health outcomes compared to current practice.

## Graphical Abstract

**Graphical Abstract:**
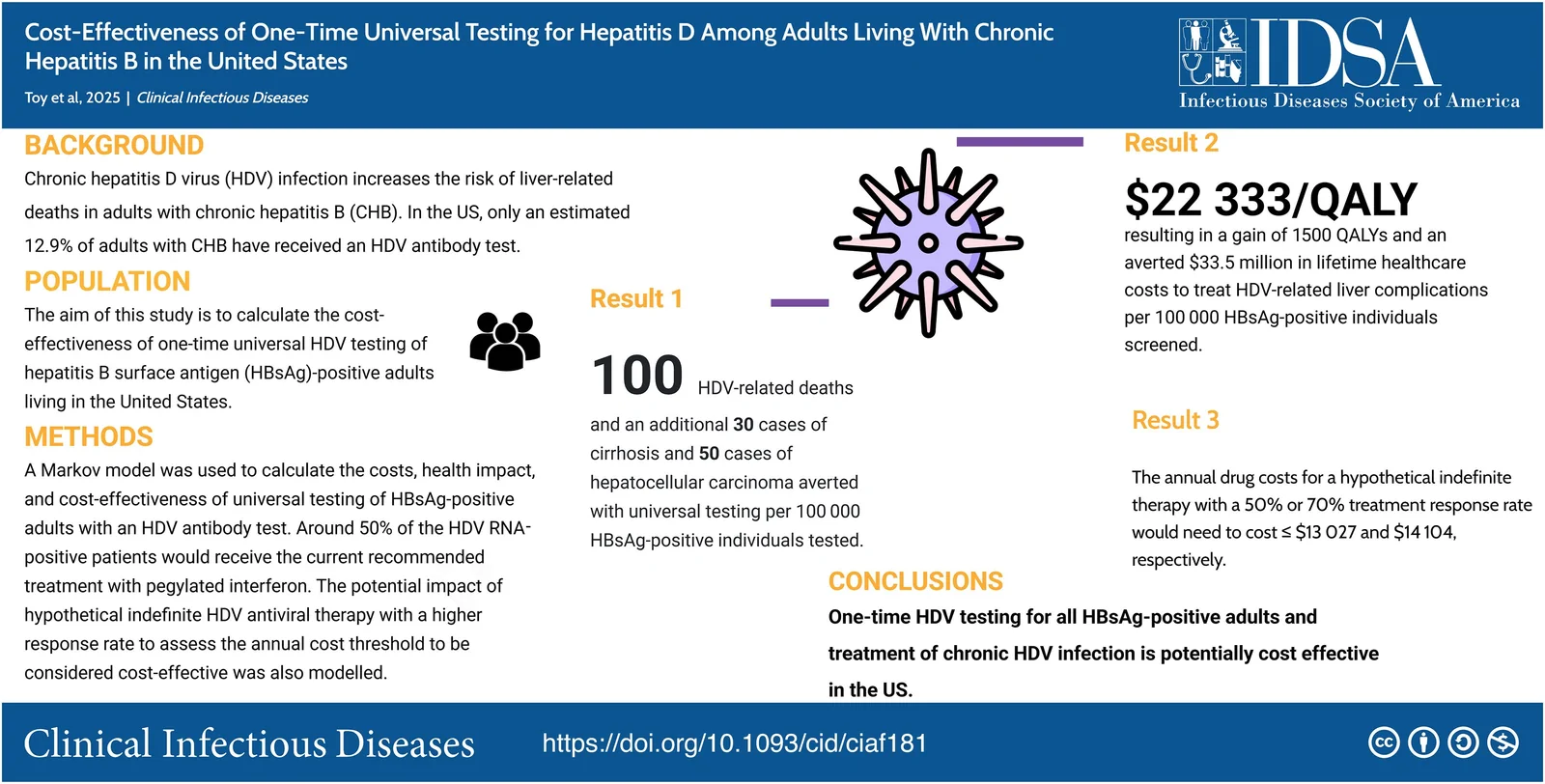
This graphical abstract is also available at Tidbit: https://tidbitapp.io/tidbits/cost-effectiveness-of-one-time-universal-testing-for-hepatitis-d-among-adults-living-with-chronic-hepatitis-b-in-the-united-states?utm_campaign=tidbitlinkshare&utm_source=IO

Hepatitis D virus (HDV) is the smallest known virus infecting mammals and requires the hepatitis B surface antigen (HBsAg) to infect hepatocytes [1]. Superinfection, or HDV infection in a person already diagnosed with chronic hepatitis B virus (HBV) infection, can lead to chronic HDV infection in 90% of individuals. Chronic HDV accelerates the course of hepatitis and can result in cirrhosis in 60%–90% of patients. The risk of hepatocellular carcinoma (HCC) is 3 times higher among patients with both HDV and HBV compared with patients with HBV monoinfection [2].

The most effective way to control HDV is to prevent HBV infection with hepatitis B vaccination since HDV is dependent on HBV for entry into hepatocytes and propagation. Although there is no United States (US) Food and Drug Administration (FDA)–approved treatment specifically for HDV infection, the American Association for the Study of Liver Diseases (AASLD) recommends that patients with chronic HDV (HDV RNA positive) who have elevated alanine aminotransferase (ALT) or compensated cirrhosis receive treatment with pegylated interferon (PEG-IFN) [3, 4]. Although PEG-IFN is reported to have only a 30% response rate with an estimated 8% relapse rate per year, patients who achieved a combined virologic and biochemical response (undetectable or at least a 2-log_10_ decrease in HDV RNA and normalization of ALT) at 24 weeks after PEG-IFN therapy had improved event-free survival [4]. However, interferon therapy is associated with many side effects [5]. Recent progress in HDV research has given rise to several new investigational drugs that may be more effective and have fewer side effects than interferon [3, 4]. Bulevirtide, a drug given daily by subcutaneous injection that blocks the entry of HDV into hepatocytes, was approved for HDV treatment by the European Medicines Agency [6].

Studies assessing the prevalence of exposure to HDV (HDV antibody [anti-HDV] positive) in the US among HBsAg-positive adults have reported widely varying results, with anti-HDV positivity ranging from 2% to 6% and as high as 41% among Mongolians living in Los Angeles [7–10]. A recent meta-analysis estimates a 3.8% HDV prevalence among HBsAg-positive persons in the US [11]. A global HDV prevalence meta-analysis by Stockdale et al [12] estimated that 64% of anti-HDV–positive adults are HDV RNA positive. Taken together, these estimates imply that as many as 2.4% (3.8% anti-HDV × 64.2% RNA positive) of the estimated 660 000 to 1.6 million HBsAg-positive adults in the US are living with chronic HDV infection [13, 14]. However, the current AASLD recommendation for HDV testing is limited to persons at high risk of HDV (defined as persons who inject drugs, those with human immunodeficiency virus [HIV] and/or hepatitis C virus [HCV] infection, men who have sex with men, those with a history of sexually transmitted infections or multiple sexual partners, immigrants from areas with high HDV endemicity, or those who have HBV DNA <2000 IU/mL but elevated ALT) [15]. The testing rate for HDV is very low, with an estimated 12.9% (range, 6.7%–19.7%) of HBsAg-positive adults having been screened for anti-HDV [16].

Recently, universal HBV screening in the US was found to be highly cost-effective [17], prompting the Centers for Disease Control and Prevention (CDC) to update its guidelines to recommend one-time hepatitis B screening for all adults aged ≥18 years [18]. The aim of this study is to calculate the cost-effectiveness of one-time hepatitis D testing among HBsAg-positive adults in the US.

## METHODS

### Overview

We developed a Markov model to simulate long-term outcomes of chronic hepatitis B (CHB) patients with HDV infection. We summarized health outcomes using quality-adjusted life years (QALYs), computed direct medical costs, and assessed cost-effectiveness using incremental cost per QALY ratios, over a lifetime horizon. A threshold of <$50 000 per QALY is generally considered cost-effective in the US [19].

### Natural History Model

The natural history model included progression rates for cirrhosis, HCC, and liver-related death. Disease progression rates of untreated chronic HDV were derived from cohort studies and meta-analyses of HDV patients (Supplementary Table 1 and Supplementary Figure 1). Patients in the model can be diagnosed depending on one of 2 strategies, described below. Diagnosed patients may start treatment, which reduces disease progression risks compared to those among untreated patients. The model captures mortality from HBV, HDV, or other causes. Mortality rates from causes other than HBV or HDV were based on life tables from US vital statistics [20]. We simulated a hypothetical cohort of diagnosed HBsAg-positive patients starting from age 45 years. Model transitions were simulated in one-year time steps. The model was implemented in TreeAge Pro 2023.

### Screening Scenarios

**Figure 1.**
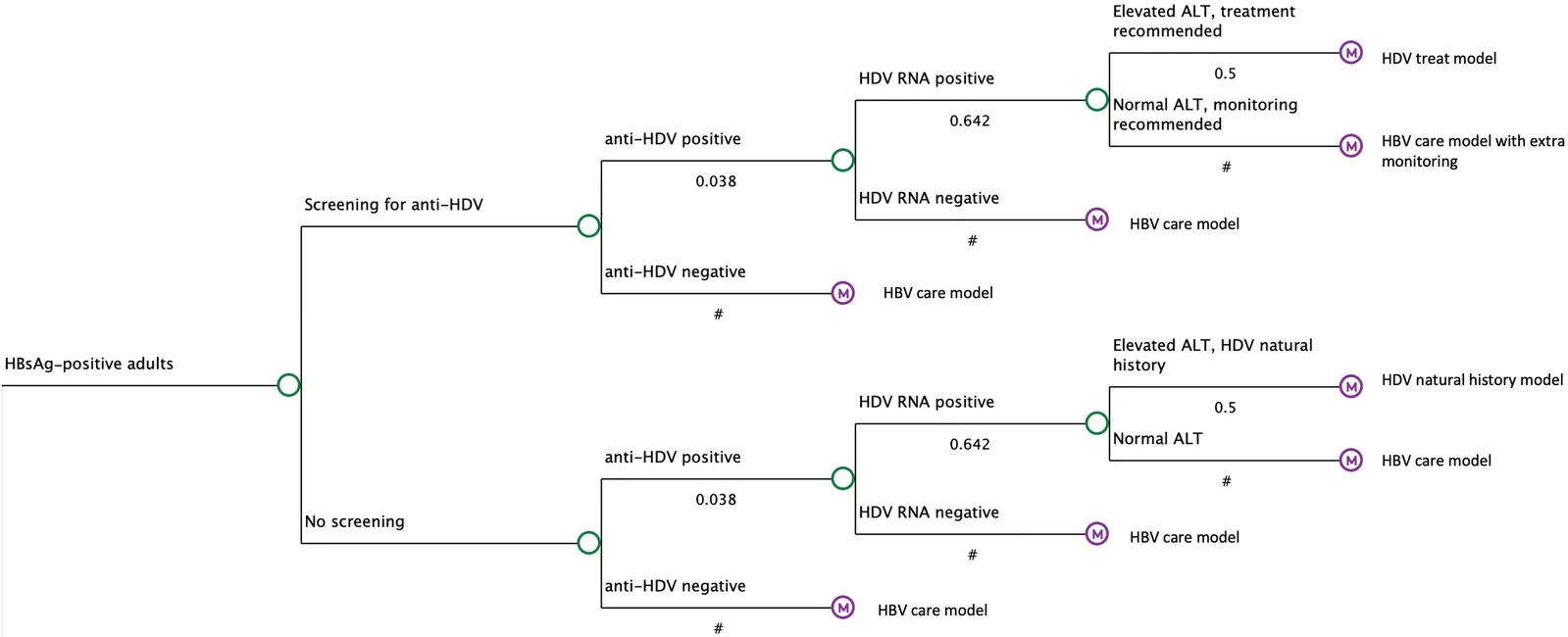
Decision tree for hepatitis D virus antibody testing and treatment vs no testing among hepatitis B surface antigen–positive adults who are aware of their infection. Abbreviations: ALT, alanine aminotransferase; anti-HDV, hepatitis D virus antibody; HBsAg, hepatitis B surface antigen; HBV, hepatitis B virus; HDV, hepatitis D virus.

We compared 2 main screening scenarios: status quo levels of testing and universal one-time testing. For the status quo, we assumed that 12.9% of the cohort had been screened for exposure to HDV with an anti-HDV test, based on current screening rates. For the universal testing scenario, we assumed that 100% of the cohort was screened for exposure (Figure 1). In both scenarios, people who tested positive for anti-HDV were then tested for HDV RNA. If the HDV RNA test was negative, the person did not have chronic HDV and those HBsAg-positive individuals followed the CHB care model, adapted from our previous studies [17]. In this model, we assumed that 3.8% of the HBsAg-positive individuals screened for exposure would test positive for anti-HDV [11]; 64.2% of the anti-HDV–positive individuals would test positive for HDV RNA, meaning a diagnosis of chronic HDV infection [12]; and 30% would have cirrhosis [21]. In this study, we assumed that only 50% of the HDV RNA–positive patients would receive HDV treatment based on having compensated cirrhosis or an elevated ALT and no contraindication for PEG-IFN. Key input variables are shown in Table 1.

**Table 1.** Key Input Variables for the Model

Variable	Base Case	Range	Reference
Age/birth cohort	45 y	18–70 y	
Male-to-female prevalence ratio	58:42	…	Patel et al, 2019 [9]
CHB adults receiving anti-HDV screening (current status)	12.9%	5%–20%	Nathani et al, 2023 [16]
Anti-HDV positivity among HBsAg-positive adults	3.8%	1.0%–10.0%	Wong et al, 2024 [11, 22]
Proportion of anti-HDV–positive adults testing positive for HDV RNA	64.2%	20%–100%	Stockdale et al, 2020 [12]
Percent of anti-HDV–positive persons with cirrhosis	30.0%	10%–50%	Kamal et al, 2022 [21]
Percent of HDV viremic patients receiving HDV treatment (elevated ALT or with compensated cirrhosis)	50.0%	20%–100%	assumption
Combined virologic and biochemical response rate with PEG-IFN alfa-2a therapy	29.0%	24%–34%	Abdrakhman et al, 2022 [5]
Combined virologic and biochemical response rate with hypothetical drug therapy	50.0%	50%–90%	assumption
Relapse rate for PEG-IFN alfa-2a per year	8.0%	8%–10%	Mentha et al, 2019 [23]
Costs of testing			
Anti-HDV test	$18.16	$17–$82	Medicare reimbursement [24]
HDV RNA test	$42.84	$35–$300	Medicare reimbursement [24]
Annual medical management costs			
Cirrhosis	$5964	$202–$7096	Liu et al, 2012 [25]
Decompensated cirrhosis	$15 795	$4901–$37 081	Liu et al, 2012 [25]
Liver transplantation first year	$215 162	$167 163–$250 746	Liu et al, 2012 [25]
Liver transplantation second year	$26 860	$23 958–$35 937	Liu et al, 2012 [25]
Costs of PEG-IFN treatment × 48 weeks			
PEG-IFN alfa-2a 180 µg/week (48 weeks) drug cost	$49 031	$49 031–$60 000	Red Book [23]
Cost of monitoring and clinic visits for 48 weeks of HDV treatment^a^	$1276	$873–$1309	Medicare reimbursement [24]
LFT and CBC with platelet count	$86.74 × 4	…	Medicare reimbursement [24]
TSH test	$16.80 × 4	…	Medicare reimbursement [16]
HDV RNA test	$42.84 × 4	…	Medicare reimbursement [24]
HBV DNA test	$59 × 4	…	Medicare reimbursement [24]
HBsAg test	$10.33 × 4	…	Medicare reimbursement [24]
Clinic visits	$74 × 8	…	Medicare reimbursement [24]
CBC	$7.77 × 12	…	Medicare reimbursement [24]
CMP	$10.56 × 4	…	Medicare reimbursement [24]
Costs of long-term monitoring of HDV RNA–positive patients^b^	$250	…	Medicare reimbursement [24]
Health state utilities			
Disutility for HDV infection	0.05	…	assumption
Utility for PEG-IFN treatment	0.77	…	Wong et al, 2000 [26]

Abbreviations: ALT, alanine aminotransferase; CBC, complete blood count; CHB, chronic hepatitis B; CMP, comprehensive metabolic panel; HBsAg, hepatitis B surface antigen; HBV, hepatitis B virus; HDV, hepatitis D virus; LFT, liver function test; PEG-IFN, pegylated interferon; TSH, thyroid-stimulating hormone.

^a^Forty-eight weeks of treatment with hypothetical drug with fewer side effects: same monitoring as PEG-IFN treatment but without TSH testing, and decreased frequency for CBC × 4 and clinic visit × 4.

^b^Long-term monitoring costs include biannual CBC, CMP, HDV RNA, HBV DNA, and abdominal ultrasound, and annual HBsAg.

### HDV Treatment

We analyzed treatment with PEG-IFN for 48 weeks with 29% combined response rate [5] and 8% relapse rate per year [27]. In a secondary analysis, we considered a hypothetical HDV antiviral treatment taken indefinitely with either a 50% or 70% initial combined response rate. Combined response is defined as a virological and biochemical response, with HDV RNA undetectable or decreased by >2 log_10_ from baseline and normal ALT levels.

### Assumptions

Diagnosis of HDV infection consisted of a one-time anti-HDV test followed when positive by an HDV RNA test. We assumed that anti-HDV–negative and HDV RNA–negative patients would have the same disease progression rate and would receive the same monitoring as for patients with CHB monoinfection [17]. The AASLD guideline recommendation is that nucleoside analogs (NAs) are indicated when control of HBV replication is appropriate [15]. We assumed that patients with cirrhosis would receive NAs tenofovir or entecavir, which are first-line treatment for HBV, regardless of whether they receive HDV treatment. We assumed that HDV RNA–positive patients who did not receive HDV treatment and treatment nonresponders and relapsers would follow HDV natural history disease progression estimates and would receive monitoring with an annual HDV RNA test and biannual liver ultrasound for liver cancer surveillance, in addition to routine CHB monitoring and treatment.

We did not include an annual rescreening test for those at ongoing risk of exposure to HDV, since the rate of acquiring HDV in the US is unknown. In this model, we assumed that only 50% of the HDV RNA–positive patients (70% of whom have elevated ALT and 30% of whom have compensated cirrhosis) would receive HDV treatment.

### Costs and Utilities

We included a Medicare reimbursement cost of $18.16 [24] for a total anti-HDV test, followed by an HDV RNA test. We also used the Medicare reimbursement cost of $42.84 for an HDV RNA test but varied this up to $300 in sensitivity analyses. We assumed that HDV testing is performed as part of routine monitoring of patients with CHB. For patients receiving PEG-IFN treatment, we assumed that the patient would receive additional monitoring that would include complete blood count, liver function tests, thyroid-stimulating hormone test, comprehensive metabolic panel, quantitative HDV RNA test, HBV DNA test, HBsAg test, and monthly clinic visits during the 48-week treatment period (Table 1). The 48-week drug cost was $49 031 for PEG-IFN [28]. All costs were adjusted to 2024 US dollars ($) using the Medical Consumer Price Index calculator. We included age-specific, per-person background medical costs using recently published US estimates [29]. All costs and QALYs were discounted at 3% per year. The analysis was performed from the healthcare system perspective. We used EuroQoL 5 Dimensions (EQ-5D) utility assessments by Woo et al [30] and included age adjustments from Parikh et al [31]. We added a disutility of 0.05 for all HDV health states, except for treatment utilities with the more effective hypothetical treatments, that could improve health-related quality of life measured by EQ-5D visual analogue scale [32]. We added a lower utility for PEG-IFN treatment due to its low tolerance rate [26]. Medical management costs such as cirrhosis, decompensated cirrhosis, HCC, and liver transplantation are all included in the model. Key input variables related to costs and utilities are shown in Table 1 and Supplementary Table 2.

### Sensitivity Analysis

We conducted one-way sensitivity analyses to determine the parameters that had the greatest impact on the results. Sensitivity analyses were performed with anti-HDV prevalence ranging from 1.0% to 10%, prevalence of HDV RNA among anti-HDV positive patients ranging from 20% to 100%, treatment rates ranging from 20% to 100%, and proportion of patients with cirrhosis who received treatment ranging from 30% to 50%. The costs of the HDV RNA test and the hypothetical more effective treatments were varied in cost-effectiveness threshold analyses. Finally, we conducted a probabilistic sensitivity analysis to evaluate the impact of overall parameter uncertainty and outcomes.

## RESULTS

### One-time HDV Testing and Treatment With PEG-IFN

Compared to current practice, where only 12.9% of adults with CHB infection in the US are screened for HDV, one-time universal testing of adults with CHB and subsequent treatment of 50% of the HDV RNA–positive patients with PEG-IFN could potentially avert 100 HDV-related deaths and an additional 30 cases of cirrhosis and 50 cases of HCC, per 100 000 HBsAg-positive individuals screened. Compared to current practice, universal testing and treatment with PEG-IFN has an incremental cost-effectiveness ratio (ICER) of $22 333/QALY, resulting in a gain of 1500 QALYs and an averted $33.5 million in lifetime healthcare costs to treat HDV-related liver complications per 100 000 HBsAg-positive individuals screened (Table 2).

**Table 2.** Costs, Cost-effectiveness, and Health Impact of Universal Hepatitis D Virus (HDV) Testing and Treatment of 50% of HDV RNA–Positive Patients With Pegylated Interferon for an Estimated 100 000 Population With Chronic Hepatitis B Compared With the Current Screening Rate of 12.9%

Scenario	Cost, Millions	QALYs	ICER^a^	HDV Cirrhosis Cases, No.	HDV Decompensated Cirrhosis Cases, No.	HDV HCC Cases, No.	HDV Deaths, No.
Natural history	$19 089	2 039 100	…	390	230	650	1060
Current practice	$19 094	2 039 300	…	390	230	640	1050
Screen all	$19 127	2 040 800	$22 333	360	210	590	950

Abbreviations: HCC, hepatocellular carcinoma; HDV, hepatitis D virus; ICER, incremental cost-effectiveness ratio; QALYs, quality-adjusted life-years.

^a^ICER is compared to current practice.

For an estimated prevalence of 0.2%, or 660 000 HBsAg-positive adults as reported by Bixler et al [13], and assuming 50% are diagnosed or aware of their HBV status [13], universal HDV testing could avert 330 HDV-related deaths and an additional 99 cases of cirrhosis and 66 cases of HCC. If the prevalence of CHB is 0.65% or 1.6 million as reported by Lim et al [14], and assuming 50% are diagnosed, universal testing could avert 240 cases of cirrhosis, 160 cases of HCC, and 800 HDV-related deaths.

### Estimated Health Impact and Cost Threshold for Hypothetical Indefinite New Therapies

**Figure 2.**
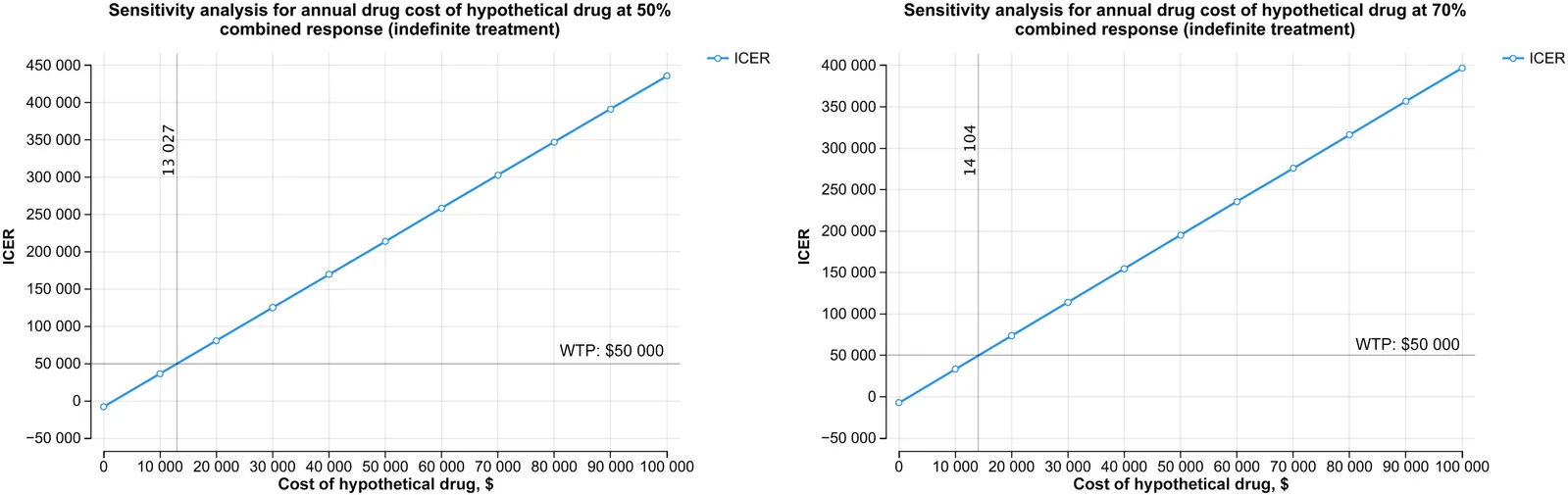
Sensitivity analysis for annual drug cost of hypothetical indefinite therapy with a 50% combined response (*A*) or 70% combined response (*B*) at a willingness-to-pay threshold of $50 000. Abbreviations: ICER, incremental cost-effectiveness ratio; WTP, willingness to pay.

Universal HDV testing and treatment of 50% of the HDV RNA–positive patients with hypothetical indefinite duration HDV antiviral therapy with a 50% or 70% combined virologic and biochemical response could avert 71% and 74% of HDV-related deaths, respectively, assuming a best case scenario of perfect treatment compliance and no drug resistance (Supplementary Tables 3–5). At a willingness-to-pay (WTP) threshold of $50 000/QALY, the annual drug cost of the indefinite therapy with a 50% or 70% combined response rate would need to be <$13 027 and <$14 104, respectively (Figure 2). The hypothetical indefinite therapy with a 50% or 70% combined response would be cost-saving if the annual drug cost is <$1757 and <$1729, respectively (Supplementary Figure 2). If the WTP threshold is $100 000/QALY, the annual drug cost of indefinite therapy with a 50% or 70% combined response rate would need to be <$24 377 and <$26 559, respectively (Supplementary Figures 3 and 4).

### Sensitivity Analysis

The results of one-way sensitivity analyses are summarized in Supplementary Figure 5. The model was sensitive to certain parameters such as the proportion of HDV RNA–positive patients who received HDV treatment, anti-HDV prevalence, HDV RNA prevalence, and the annual cost of the hypothetical therapies. We ran separate sensitivity analyses for each of these variables and for each treatment strategy. Varying the anti-HDV prevalence between 1% and 10% had a small impact on the ICER (Supplementary Figure 6). If anti-HDV prevalence is only 1%, universal testing and treatment with PEG-IFN would still be cost-effective at an ICER of $22 333/QALY. Varying the HDV RNA prevalence between 20% and 100% similarly had a small impact on the ICER (Supplementary Figure 7). Varying the proportion of HDV RNA–positive patients who received treatment (fraction treated) from 20% to 100% did not have a large impact on the ICER (Supplementary Figure 8), and varying the portion of patients who received treatment with cirrhosis from 30% to 50% reduced the ICER slightly to $19 587/QALY. The sensitivity analysis of hypothetical drug response rate is presented in Supplementary Figure 9. The probabilistic sensitivity analysis showed a >99% likelihood that universal screening strategies would be cost-effective at a WTP threshold of $50 000/QALY (Supplementary Figure 10).

## DISCUSSION

With an estimated anti-HDV positivity rate of 3.8% and an HDV RNA positivity rate of 64.2% among the estimated 66 000–1.6 million people living with CHB in the US, chronic HDV may affect 16 000–40 000 of Americans [11, 12, 33]. Many adults with chronic HDV likely have not been diagnosed since only a small fraction of HBsAg-positive adults in the US (∼13%) have been tested for HDV, given that testing is currently recommended by the AASLD only for persons at increased risk of HDV infection [4, 15]. The reported low HDV risk-based testing rate is not surprising since most primary care clinicians would likely not remember a long list of risk factors that are often not collected or documented in the health records, and many may not be aware of the recommendations for HDV testing. Universal anti-HDV testing of people with CHB is recommended by the European Association for the Study of the Liver, the Asian Pacific Association for the Study of the Liver, and the updated 2024 World Health Organization guidelines [3]. With the recognition that chronic HDV is underdiagnosed and with emerging new therapies, many US experts are advocating for universal HDV testing among HBsAg-positive persons [33, 34]. This study found that one-time universal HDV testing among HBsAg-positive persons and treatment of an estimated 50% of persons with chronic HDV with elevated ALT or compensated cirrhosis with PEG-IFN in the US cost $22 333/QALY. At a WTP of $50 000/QALY, the annual drug costs for a hypothetical indefinite HDV antiviral therapy with 50% or 70% combined response rate would need to cost ≤$13 027 and ≤$14 104, respectively. Since many HBV-infected individuals are unaware of their infection, there is a missed opportunity for significant improvements, including the diagnosis of HDV infections, if HBV infection ascertainment is not achieved. The potential benefits are substantial if more individuals become aware of their HBV status.

There are a number of limitations in this study. The natural history of chronic HDV infection is not well studied. In the model, disease progression rates in patients with chronic HDV are based on estimates from available published studies. Our model also assumes standard background mortality, but risk factors for HDV coinfection are correlated with behaviors that limit life expectancy. To the extent individuals with HDV have higher background mortality, we may overestimate long-term health gains. A limitation in our study is the estimated proportion of 64% anti-HDV–positive adults testing positive for HDV RNA, which was taken from a meta-analysis by Stockdale et al [12], which may not be representative of CHB among persons in the US. Knowing this limitation, we ran a sensitivity analysis around the proportion of anti-HDV–positive adults testing positive for HDV RNA that ranged from 20% to 100%. We assume that patients who are anti-HDV positive but HDV RNA negative have resolved infection and would not require further HDV RNA monitoring and would have disease progression rates similar to CHB patients without HDV. Currently there are no FDA-approved anti-HDV and HDV RNA assays, and the sensitivity and specificity of the current tests are variable and are not assessed in the study. We assumed that 50% of the HDV RNA–positive adults would not receive treatment because they either have normal ALT or decompensated cirrhosis. But treatment rates are uncertain, particularly if individuals are reluctant to take PEG-IFN or the prescribed treatment. However, treatment rates of 20%–100% were assessed in sensitivity analyses, and universal testing was still cost-effective. Although patients on bulevirtide have a 50% initial combined virologic and biochemical response rate, it would likely have to be taken long-term since most patients may relapse after stopping treatment. It is unclear whether a combination of new therapies with or without PEG-IFN will prove to be more effective [3]. Another limitation of our study is that it does not include out-of-pocket costs to patients.

In 2023, CDC recommended a one-time universal screening of all adults for HBV infection [18]. A national recommendation for universal testing for HDV among persons with HBV would require a call to action to raise clinician and public awareness.

## Supplementary Material

ciaf181_Supplementary_Data

## Supplementary Data

Supplementary materials are available at *Clinical Infectious Diseases* online. Consisting of data provided by the authors to benefit the reader, the posted materials are not copyedited and are the sole responsibility of the authors, so questions or comments should be addressed to the corresponding author.
