# Supplementary material for: Cost-Effectiveness of One-time Universal Testing for Hepatitis D Among Adults With Chronic Hepatitis B in the United States
Source: Clin Infect Dis. 2025 Apr 8;81(4):e211–7. doi: 10.1093/cid/ciaf181 (PMC12598672; doi:10.1093/cid/ciaf181)
Supplement: ciaf181_Supplementary_Data [file ciaf181_supplementary_data.docx]

**Appendix: Cost-Effectiveness of One-Time Universal Screening for Hepatitis D among Adults living with Chronic Hepatitis B in the United States**

Table of Contents

[Table 1. Disease transition estimates for HDV 2](#_Toc187231607)

[Table 2. Cost-effectiveness and clinical outcomes of universal HDV screening and indefinite treatment with a hypothetical drug with a 50% combined response 4](#_Toc187231608)

[Table 3. Cost-effectiveness and clinical outcomes of universal HDV screening and indefinite treatment with a hypothetical drug with a 70% combined response 5](#_Toc187231609)

[Table 4. Estimated life-time costs, QALYs and clinical outcomes for a population of 10,000 HDV RNA- positive patients who received indefinite treatment with a hypothetical drug 6](#_Toc187231610)

[Figure 1. Model disease progression rates in adults with untreated viremic HDV infection (denoted by an asterisk) compared with adults who had a combined virologic and biochemical response after HDV treatment 7](#_Toc187231611)

[Figure 2. Sensitivity analysis for annual drug cost of hypothetical drug with a A) 50% and B) 70% combined response at a willingness to pay threshold of $0 (cost-saving threshold) 8](#_Toc187231612)

[Figure 3. Sensitivity analysis for annual drug cost of hypothetical drug with a A) 50% and B) 70% combined response at a willingness to pay threshold of $50,000 10](#_Toc187231613)

[Figure 4. Sensitivity analysis for annual drug cost of hypothetical drug with a A) 50% and B) 70% combined response at a willingness to pay threshold of $100,000 12](#_Toc187231614)

[Figure 5. Tornado diagram, at a willingness to pay (WTP)of $50,000 14](#_Toc187231615)

[Figure 6. Sensitivity analysis of anti-HDV prevalence for treatment with (A) PEG-IFN (B) hypothetical drug with 50% combined rate 15](#_Toc187231616)

[Figure 7. Sensitivity analysis of HDV RNA-positivity prevalence for (A) PEG-IFN treatment (B) hypothetical drug with 50% combined rate 17](#_Toc187231617)

[Figure 8. Sensitivity analysis of fraction treated among HDV RNA-positive for (A) Peg-IFN treatment (B) hypothetical drug with 50% combined rate 19](#_Toc187231618)

[Figure 9. Sensitivity analysis of hypothetical drug response rate 21](#_Toc187231619)

[Figure 10. Cost-effectiveness acceptability curve 22](#_Toc187231620)

# Table 1. Disease transition estimates for HDV

| Transition natural history (per year) | **Annual transition estimates (range)** | Reference |
| --- | --- | --- |
| From HDV (viremic) |  |  |
| Compensated Cirrhosis | 3.70% (2.96-4.44) | Kamal et al. 2022 [1] |
| HCC | 3.00% (2.40-3.60) | Alfaiate et al. 2020 [2] |
| Death | 0.41% (0.32-0.49) | Kamal et al. 2022 [1] |
| From HDV Compensated Cirrhosis |  |  |
| Decompensated Cirrhosis | 3.90% (3.12-4.68) | Lin et al. 2005 [3]; Wranke et al. 2023 [4] |
| HCC | 3.16% (2.53-3.79) | Thiele et al. 2014 [5]; Wranke et al. 2023 [4] |
| Death | 4.89% (3.92-5.86) | Thiele et al. 2014 [5] |
| From HDV Decompensated Cirrhosis |  |  |
| Liver Transplantation | 1.20% (1.00-3.00) | OPTN [6] |
| HCC | 7.00% (3.50-10.60) | Lin et al. 2005 [3] |
| Death | 15.00% (7.50-22.50) | Lin et al. 2005 [3] |
| From HCC |  |  |
| Liver Transplantation | 7.00% (5.00-9.00) | OPTN [6] |
| Death | 42.50% | Ding et al. 2021 [7] |
| From Liver Transplantation Decomp Cirrhosis |  |  |
| Death year 1 | 17.00% (8.50-48.00) | Burra et al. 2013 [8] |
| Death year 2+ | 2.50% (1.25-24.00) | Burra et al. 2013 [8] |
| From Liver Transplantation HCC |  |  |
| Death year 1 | 16.00% (8.00-48.00) | Burra et al. 2013 [8] |
| Death year 2+ | 2.00% (2.00-25.00) | Burra et al. 2013 [8] |
| Annual transition estimates in patients with virologic response to HDV treatment |  |  |
| From HDV patients without cirrhosis who achieved virological suppression |  |  |
| Compensated cirrhosis | 0.02 | Assumption |
| HCC | 0.06% | Nguyen et al. 2019 [9] Papatheodoridis et al. 2015 [10] |
| Relapse | 8% for PEG-IFN  0% for hypothetical  infinite therapy | Zollner et al. 2022 [11] |
| From HDV patients with compensated cirrhosis who achieved virological suppression |  |  |
| Decompensated cirrhosis | 0.05% | Assumption |
| HCC | 0.40% | Nguyen et al. 2019 [9] Papatheodoridis et al. 2015 [10] |
| Death HDV | 1.20% | Nguyen et al. 2019 [9]  Papatheodoridis et al. 2015 [10] |
| Relapse | 8% for PEG-IFN  0% for hypothetical infinite therapy | Zollner et al. 2022 [11] |

# Table 2. Cost-effectiveness and clinical outcomes of universal HDV screening and indefinite treatment with a hypothetical drug with a 50% combined response

| Scenario | Cost (billions) | QALYs | ICER* | HDV Cirrhosis cases | HDV  Decompensated  Cirrhosis cases | HDV HCC cases | HDV deaths |
| --- | --- | --- | --- | --- | --- | --- | --- |
| Natural History | $19.09 | 2,039,100 | - | 390 | 230 | 650 | 1,060 |
| Current Practice | $19.13 | 2,039,600 | - | 350 | 200 | 580 | 950 |
| Screen all | $19.40 | 2,043,000 | $81,765 | 60 | 40 | 100 | 170 |

Abbreviations: HCC: Hepatocellular Carcinoma, HDV: Hepatitis D Virus, ICER: Incremental Cost-Effectiveness Ratio, QALY: Quality-Adjusted Life-year

* ICER is compared to current practice

# Table 3. Cost-effectiveness and clinical outcomes of universal HDV screening and indefinite treatment with a hypothetical drug with a 70% combined response

| Scenario | Cost (billions) | QALYs | ICER* | HDV Cirrhosis cases | HDV Decompensated cirrhosis cases | HDV HCC cases | HDV deaths |
| --- | --- | --- | --- | --- | --- | --- | --- |
| Natural History | $19.09 | 2,039,100 | - | 390 | 230 | 650 | 1,060 |
| Current Practice | $19.13 | 2,039,600 | - | 350 | 200 | 570 | 940 |
| Screen all | $19.41 | 2,043,500 | $73,410 | 40 | 30 | 80 | 130 |

Abbreviations: HCC: Hepatocellular Carcinoma, HDV: Hepatitis D Virus, ICER: Incremental Cost-Effectiveness Ratio, QALY: Quality-Adjusted Life-year

* ICER is compared to current practice

# Table 4. Estimated life-time costs, QALYs and clinical outcomes for a population of 10,000 HDV RNA- positive patients who received indefinite treatment with a hypothetical drug

| **Scenario** | **Cost (billions)** | **QALYs** | **ICER** | **HDV Cirrhosis cases** | **HDV Decompensated cirrhosis cases** | **HDV HCC cases** | **HDV deaths** |
| --- | --- | --- | --- | --- | --- | --- | --- |
| **Natural History** |  |  |  |  |  |  |  |
| No cirrhosis | $ 1.50 | 101,089 | - | 3,148 | 911 | 4,035 | 5,669 |
| Cirrhosis | $ 0.52 | 23,550 | - | - | 912 | 1,148 | 2,813 |
| All (70-30) | $ 2.02 | 124,637 | - | 3,148 | 1,823 | 5,182 | 8,482 |
| **Treat All HDV** |  |  |  |  |  |  |  |
| No cirrhosis |  |  |  |  |  |  |  |
| *50% response** | $ 3.40 | 117,152 | $ 118,298 | 441 | 137 | 579 | 813 |
| *70% response* | $ 3.45 | 119,679 | $ 104,734 | 327 | 101 | 430 | 604 |
| Cirrhosis |  |  |  |  |  |  |  |
| *50% response** | $ 1.15 | 37,788 | $ 43,991 | - | 183 | 231 | 555 |
| *70% response* | $ 1.17 | 39,222 | $ 41,726 | - | 138 | 175 | 421 |
| All (70-30)** |  |  |  |  |  |  |  |
| *Hypo 50% response** | $ 4.55 | 154,859 | $ 83,594 | 440 | 319 | 810 | 1,368 |
| *Hypo 70% response* | $ 4.62 | 158,860 | $ 75,997 | 327 | 240 | 604 | 1,025 |

* Hypothetical drug requires indefinite treatment, with 50% initial combined response and 1% relapse rate per year. Still on treatment if combined response occurs. Still at risk for developing HCC in viral response health state and can lose HBsAg. Base case cost of $20,000 per year for hypothetical drug

** The analysis is done for no cirrhosis and cirrhosis subgroups as well as the full population of All combined HDV RNA+ (where 70% of them are no cirrhosis and 30% are with cirrhosis)

The comparator for the ICER is natural history (no treatment)

# Figure 1. Model disease progression rates in adults with untreated viremic HDV infection (denoted by an asterisk) compared with adults who had a combined virologic and biochemical response after HDV treatment

* Treatment

Relapse occurs at a rate of 1-8% (depending on the treatment)

Decom. Cirr: Decompensated Cirrhosis

HCC: Hepatocellular Carcoinoma

HDV: Hepatitis D Virus

# Figure 2. Sensitivity analysis for annual drug cost of hypothetical drug with a A) 50% and B) 70% combined response at a willingness to pay threshold of $0 (cost-saving threshold)

A)


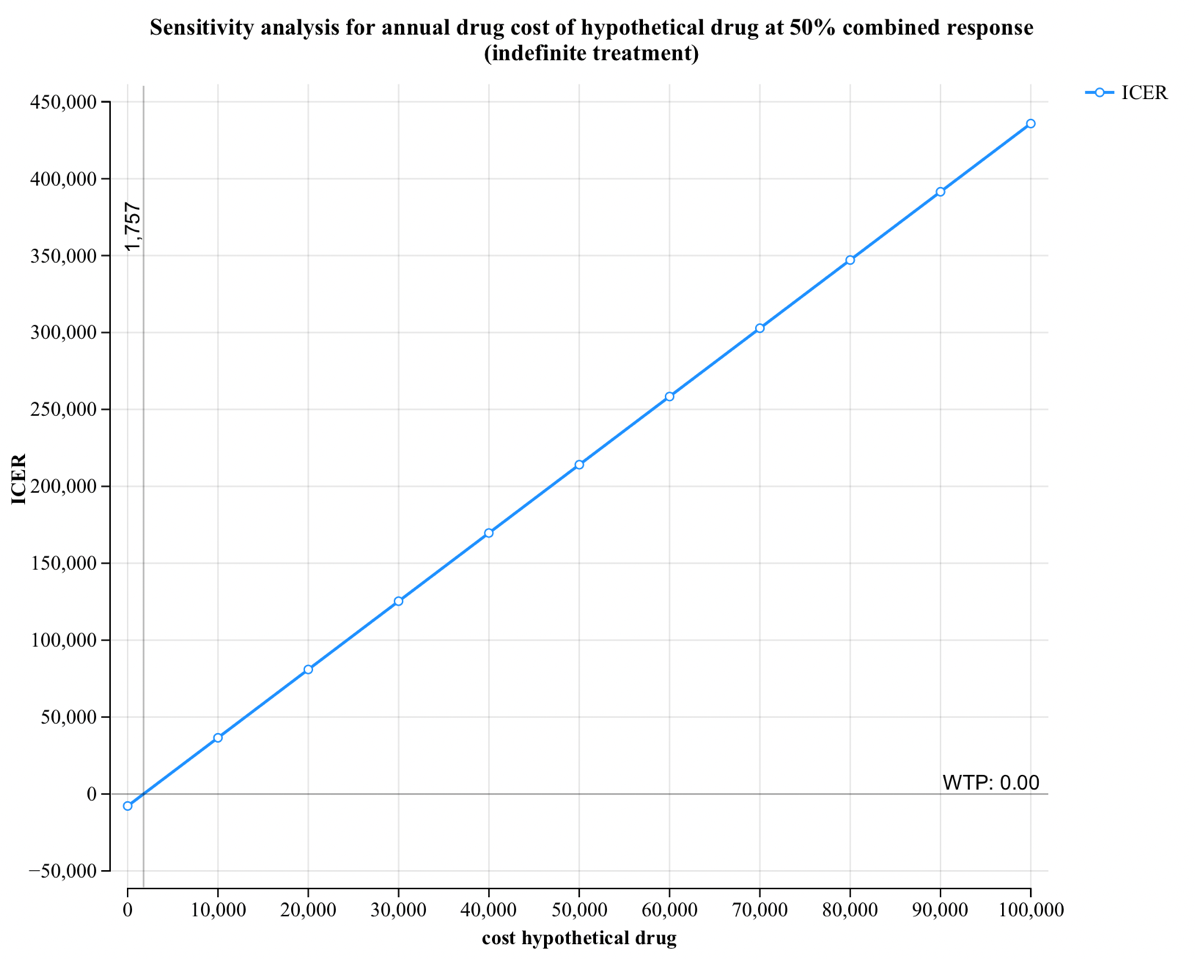


ICER, incremental cost-effectiveness ratio; WTP, willingness to pay

B)


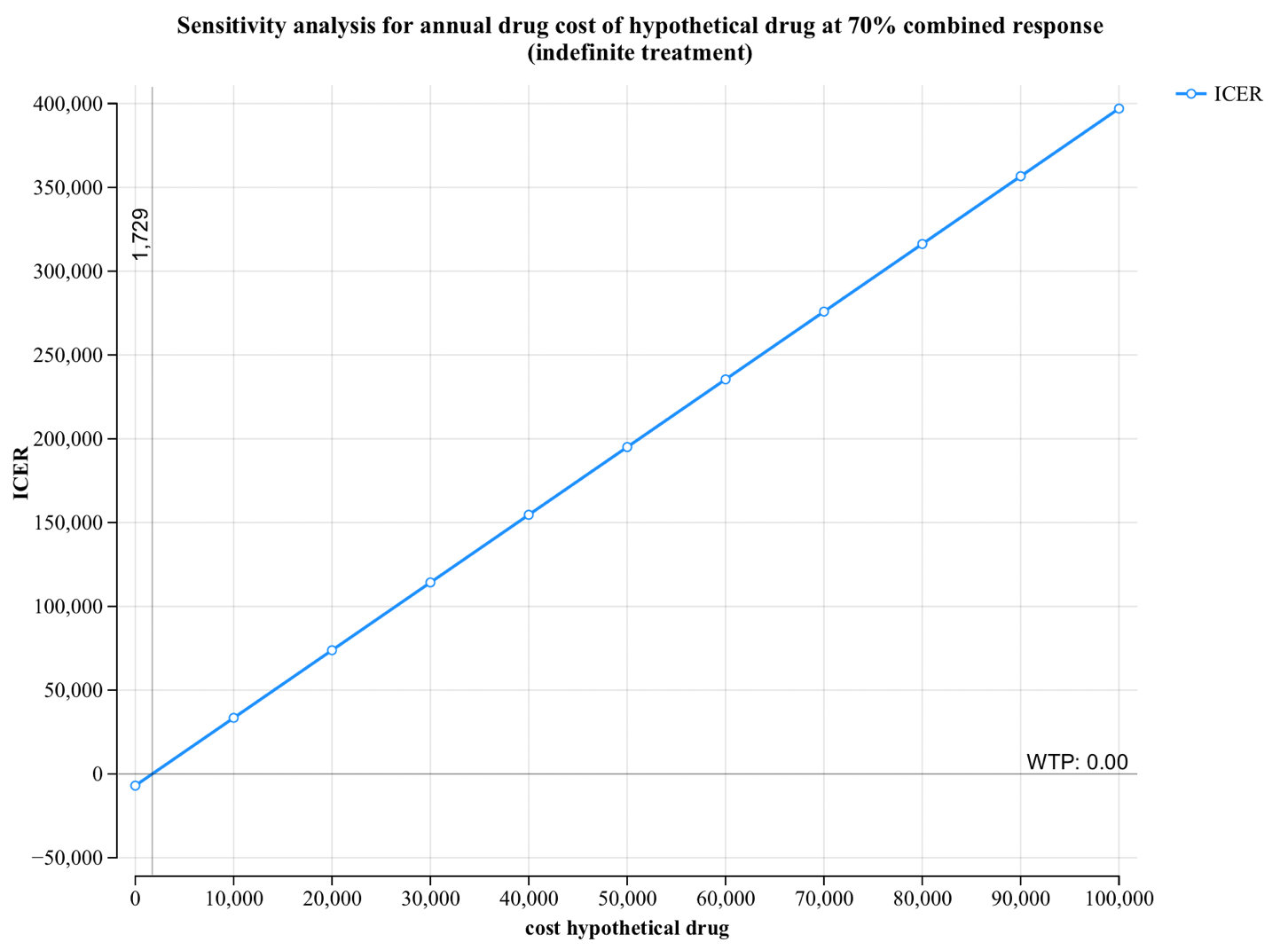


ICER, incremental cost-effectiveness ratio; WTP, willingness to pay

# Figure 3. Sensitivity analysis for annual drug cost of hypothetical drug with a A) 50% and B) 70% combined response at a willingness to pay threshold of $50,000

A)


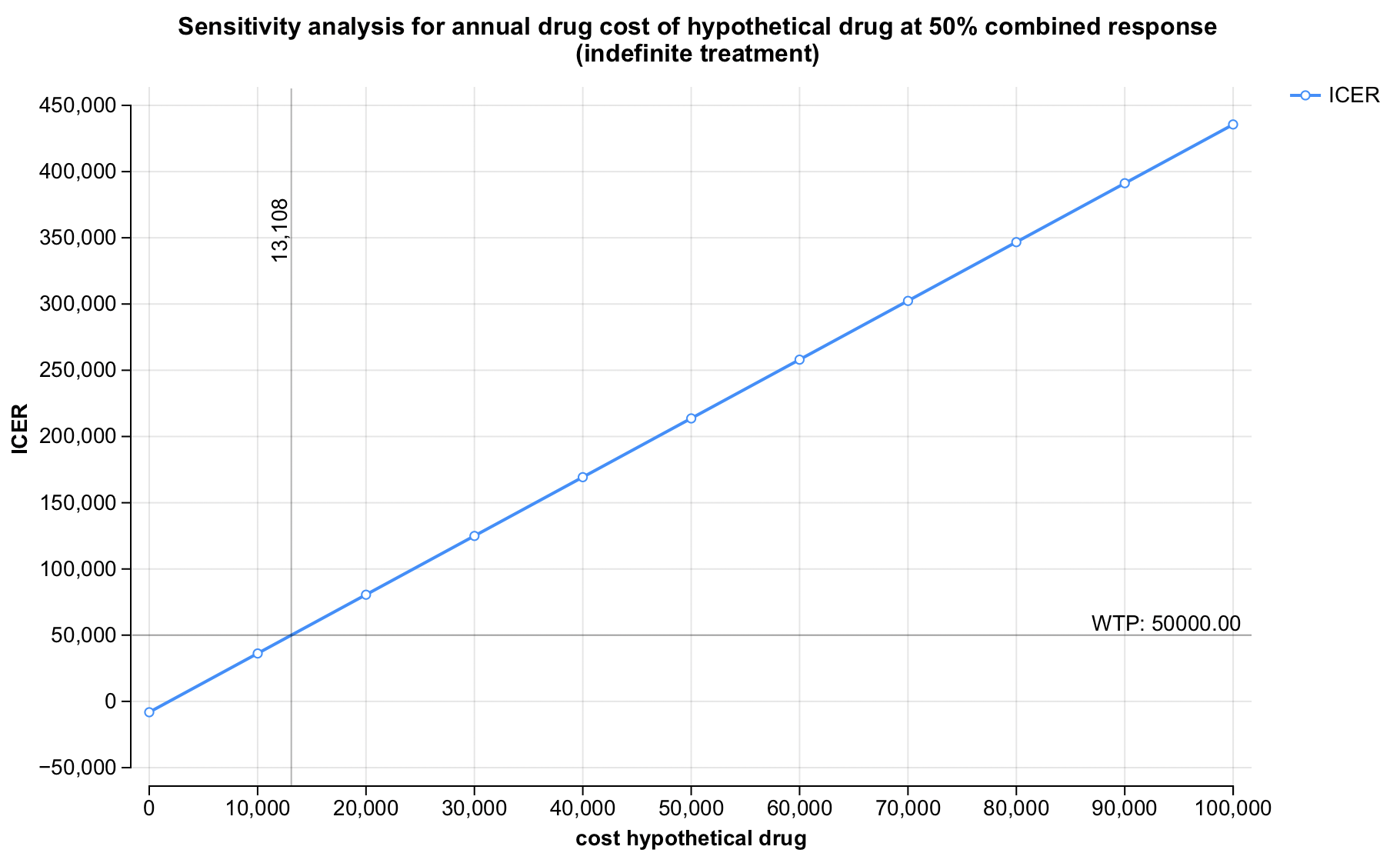


ICER, incremental cost-effectiveness ratio; WTP, willingness to pay

B)


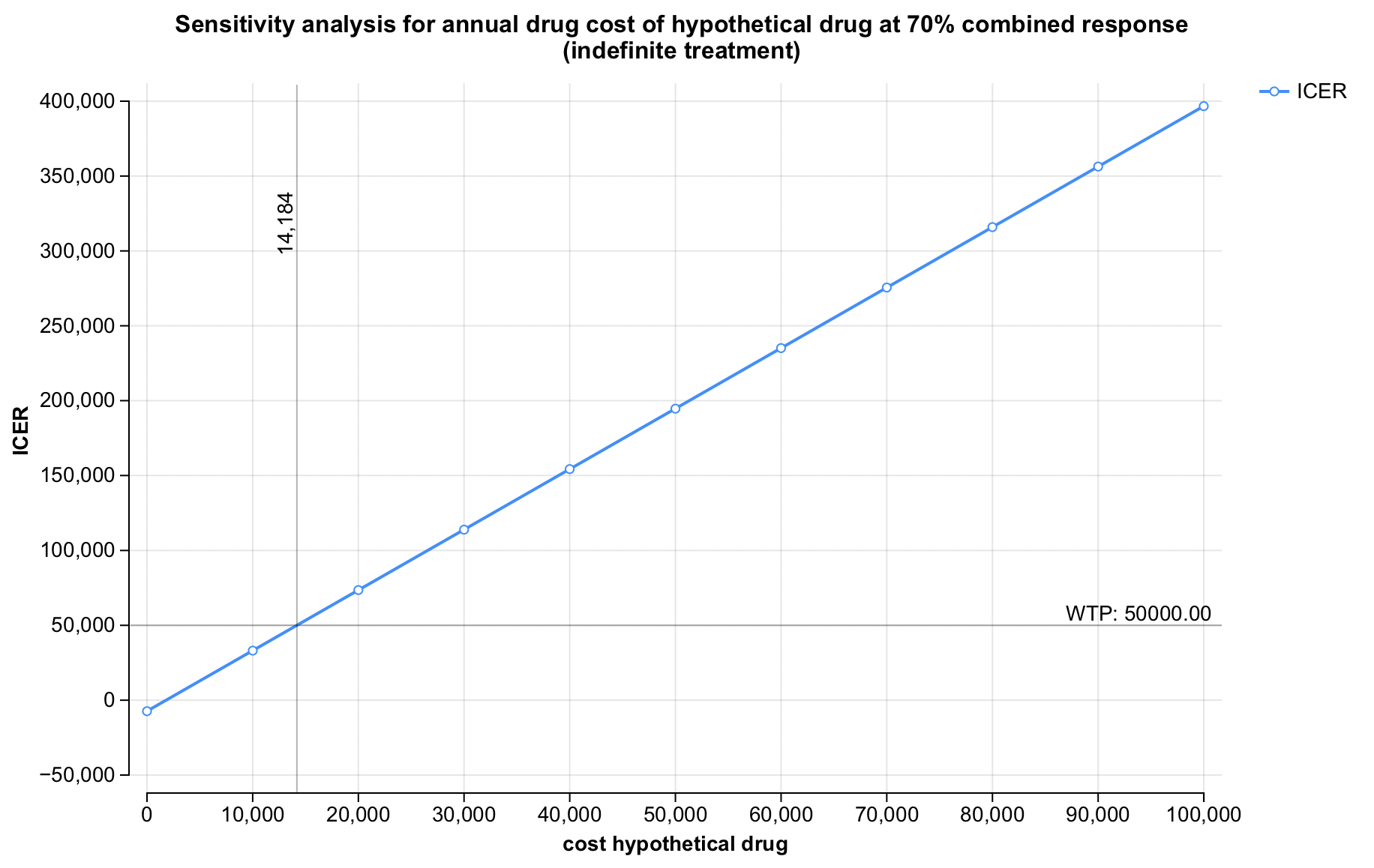


ICER, incremental cost-effectiveness ratio; WTP, willingness to pay

# Figure 4. Sensitivity analysis for annual drug cost of hypothetical drug with a A) 50% and B) 70% combined response at a willingness to pay threshold of $100,000

A)


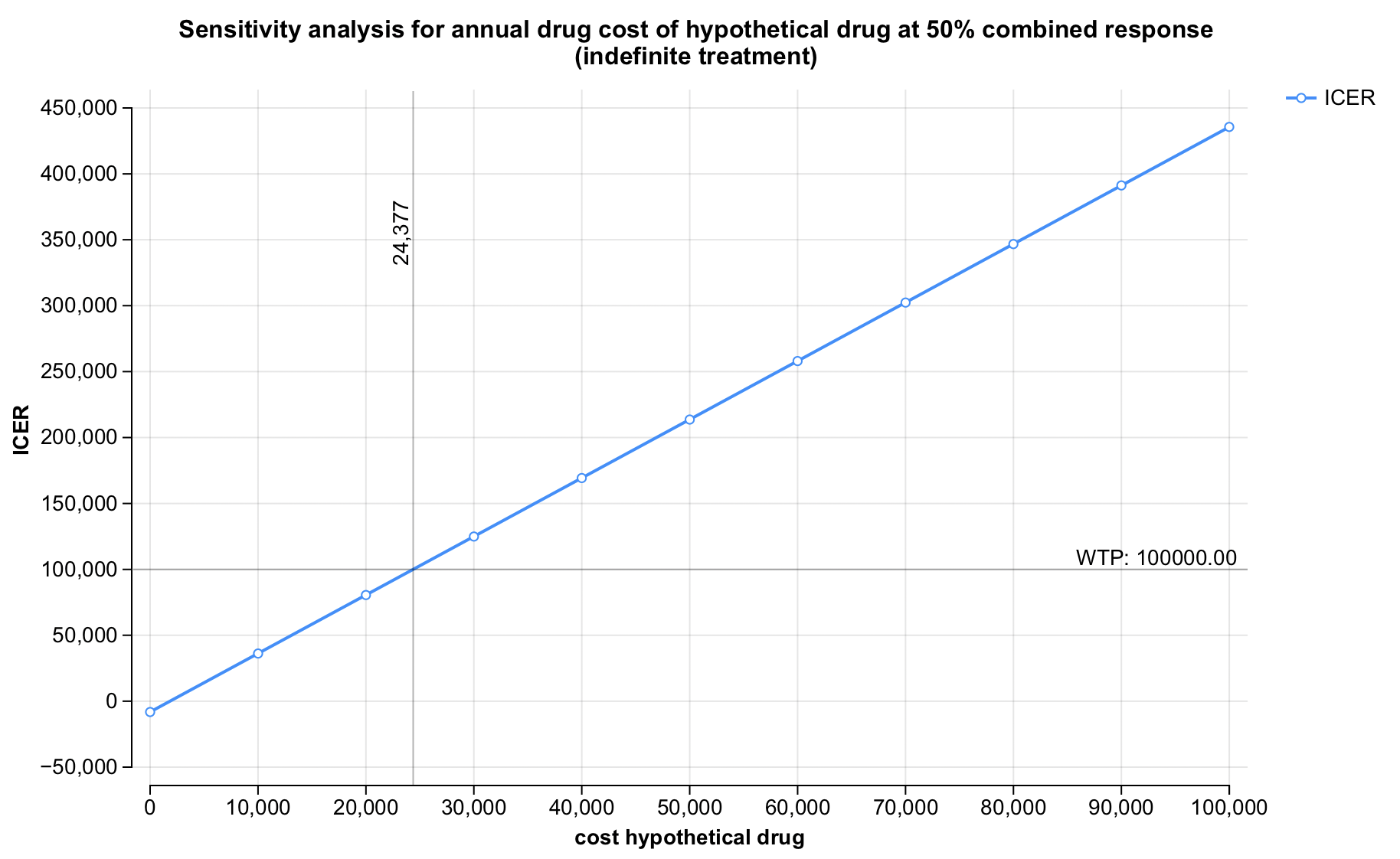


ICER, incremental cost-effectiveness ratio; WTP, willingness to pay

B)


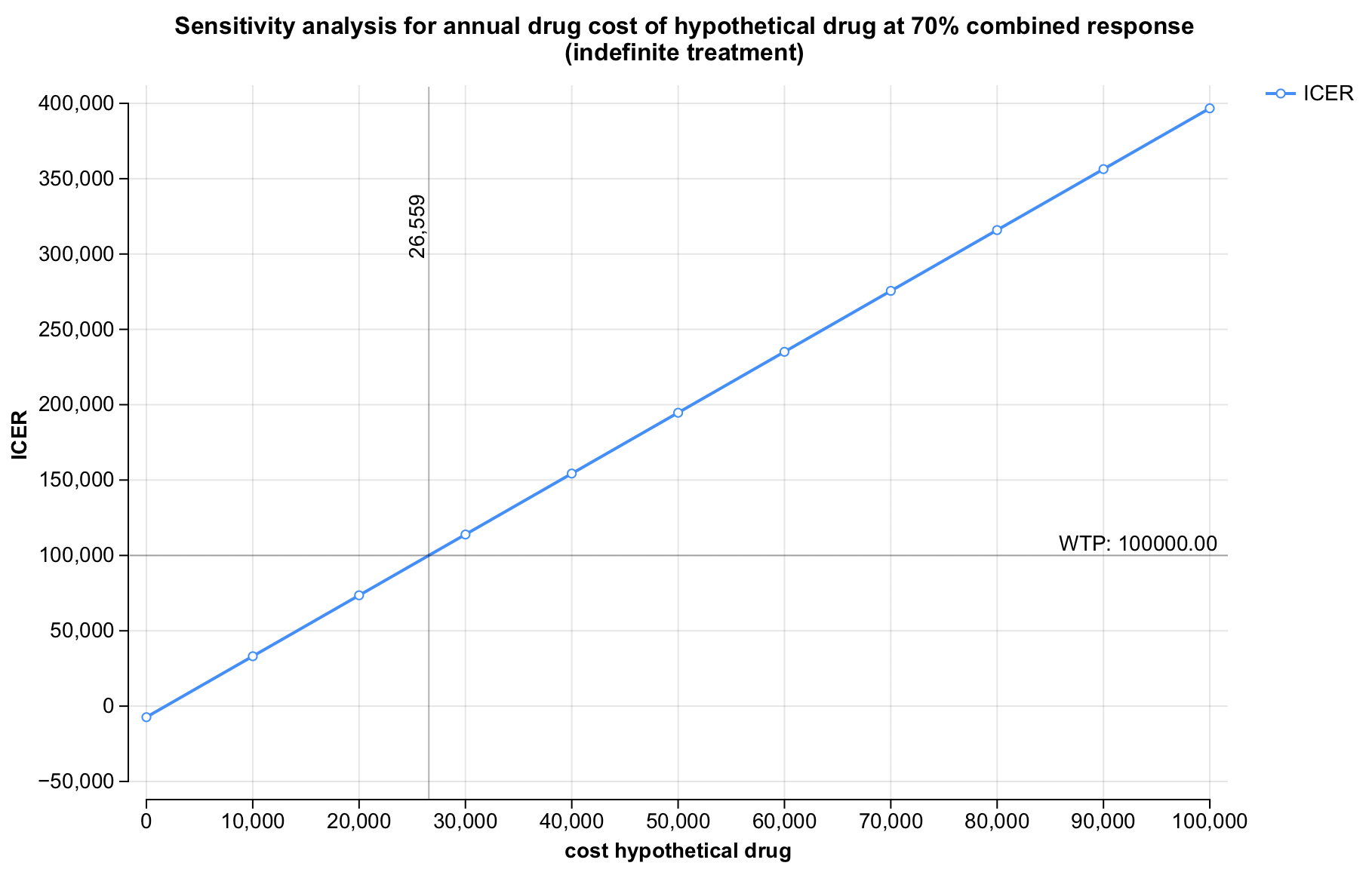


ICER, incremental cost-effectiveness ratio; WTP, willingness to pay

#

# Figure 5. Tornado diagram, at a willingness to pay (WTP)of $50,000


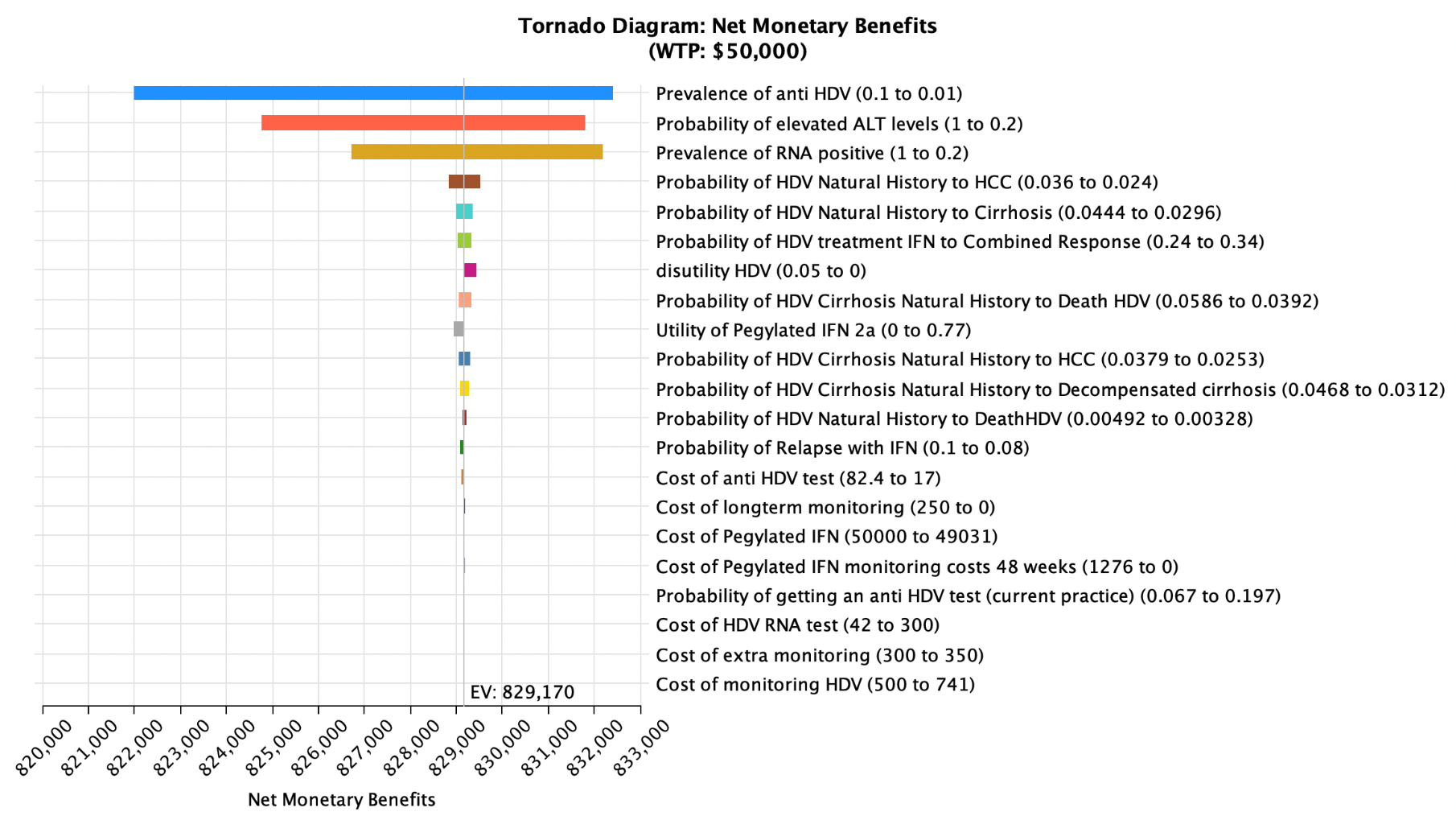


HDV: Hepatitis D Virus

Net Monetary Benefit, which is a monetary value of screening compared to no screening. This value combines both costs and QALYs together by valuing QALYs at a monetary value of $100,000 each and subtracting from that the costs. A positive NMB means screening is preferred to no screening.

#

# Figure 6. Sensitivity analysis of anti-HDV prevalence for treatment with (A) PEG-IFN (B) hypothetical drug with 50% combined rate

A)


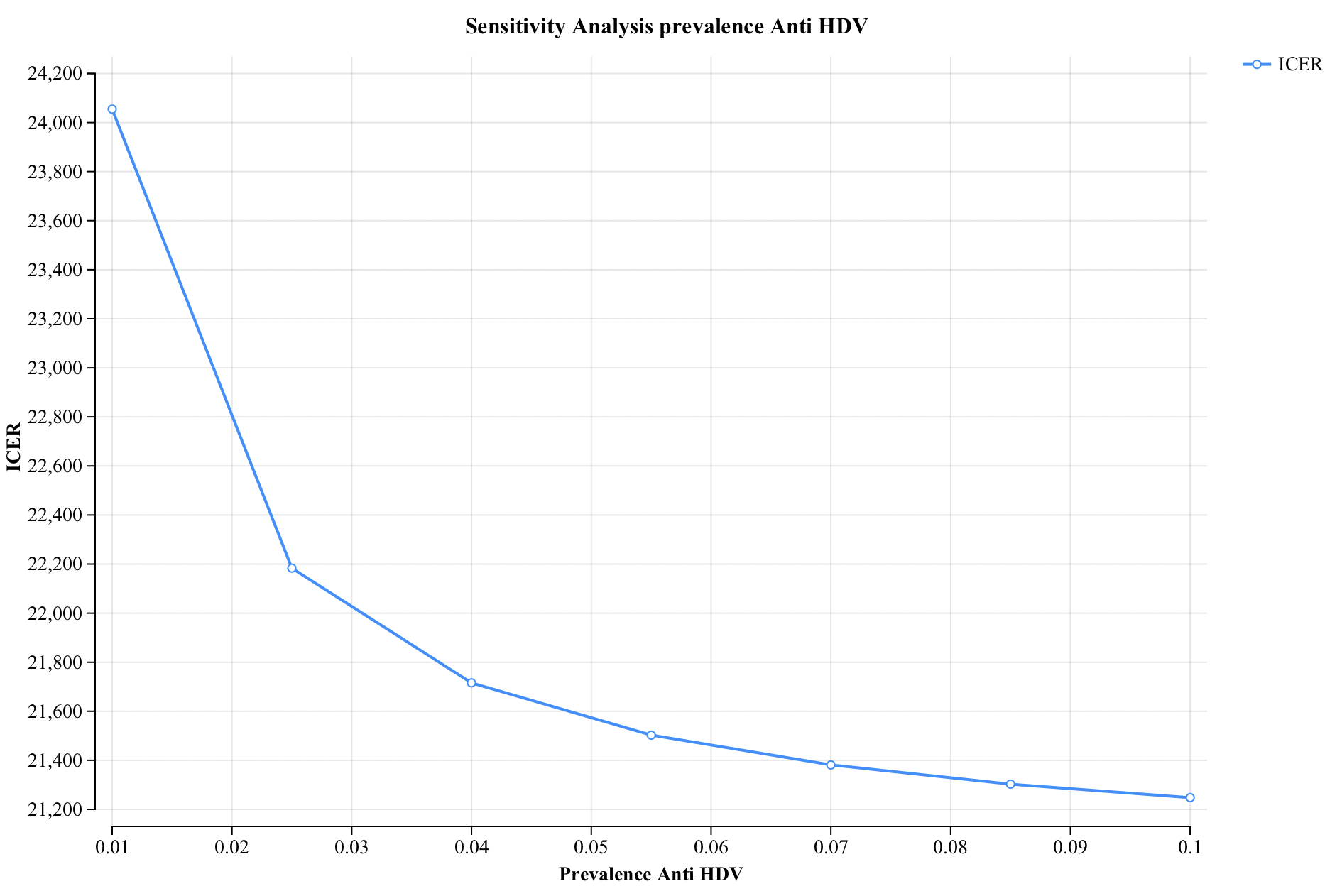


B)


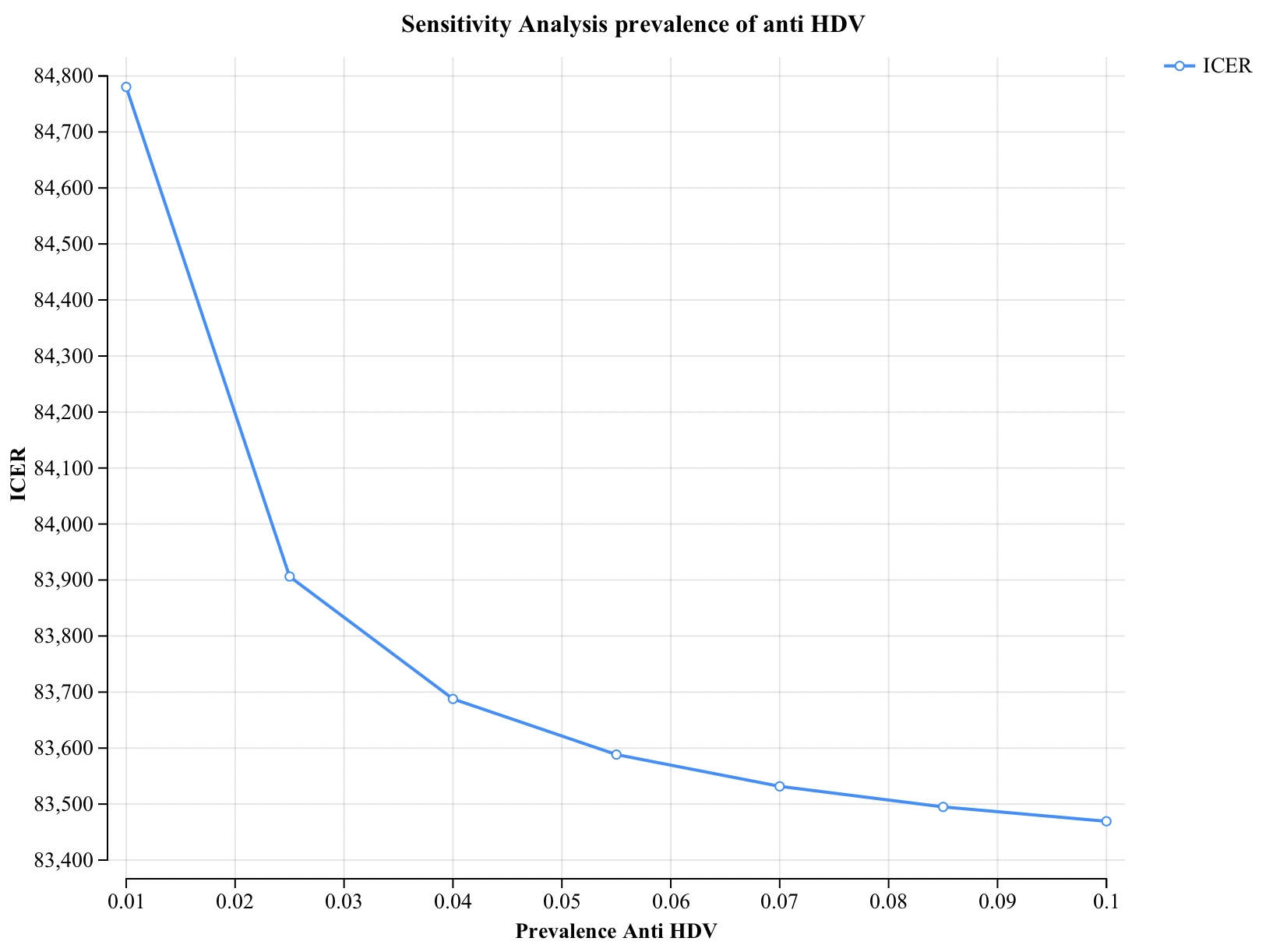


# Figure 7. Sensitivity analysis of HDV RNA-positivity prevalence for (A) PEG-IFN treatment (B) hypothetical drug with 50% combined rate

A)


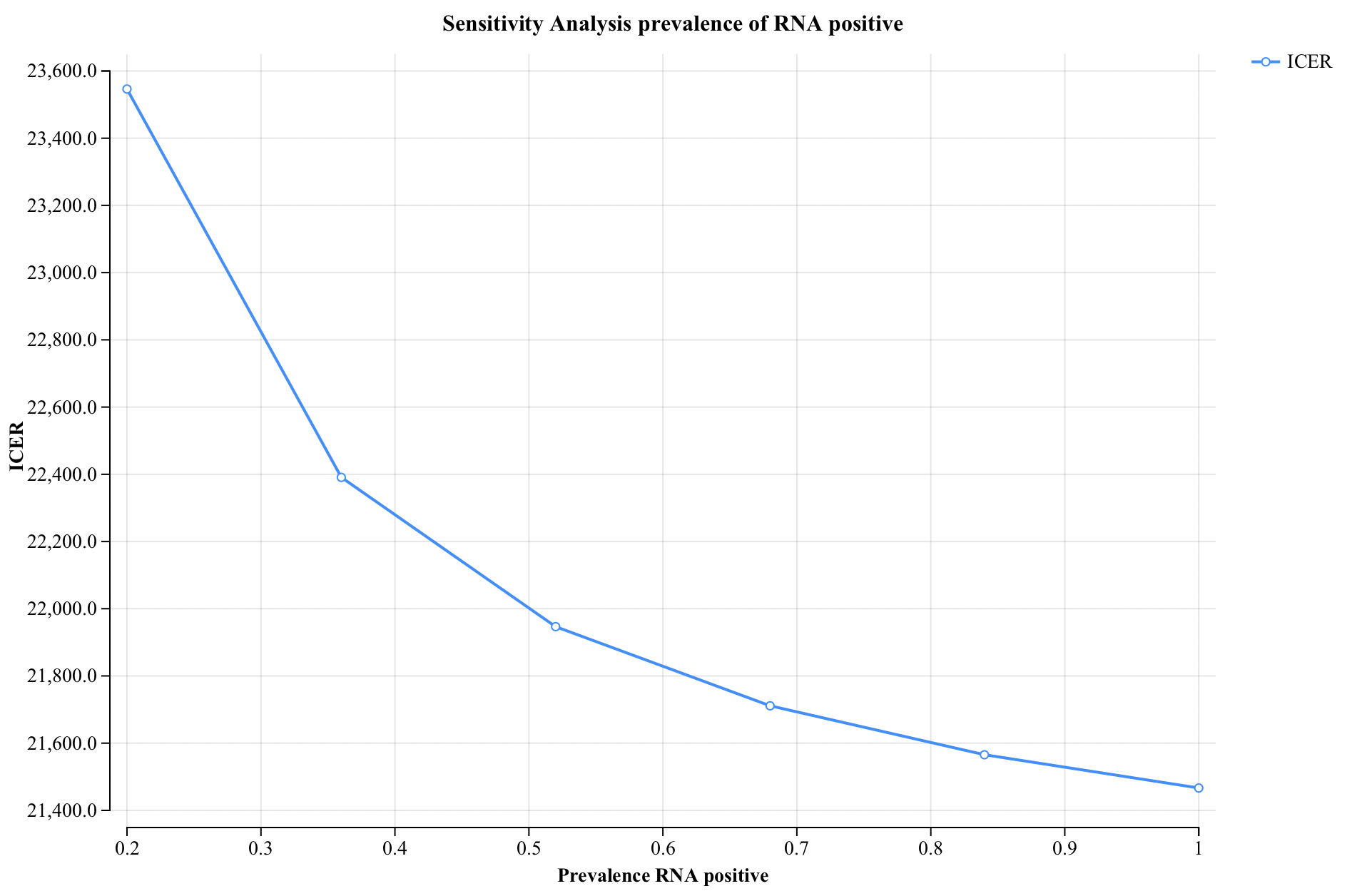


B)


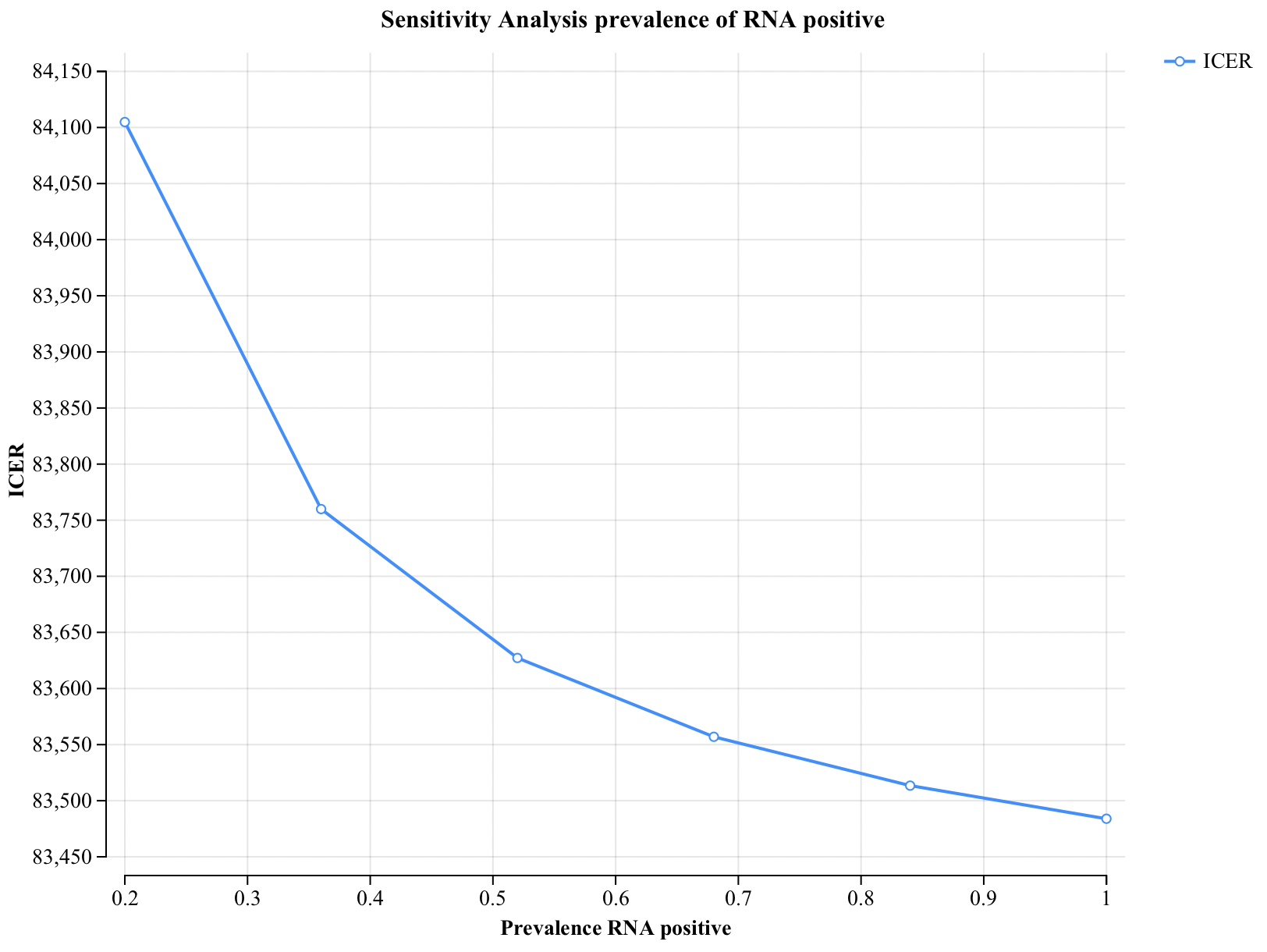


# Figure 8. Sensitivity analysis of fraction treated among HDV RNA-positive for (A) Peg-IFN treatment (B) hypothetical drug with 50% combined rate

A)


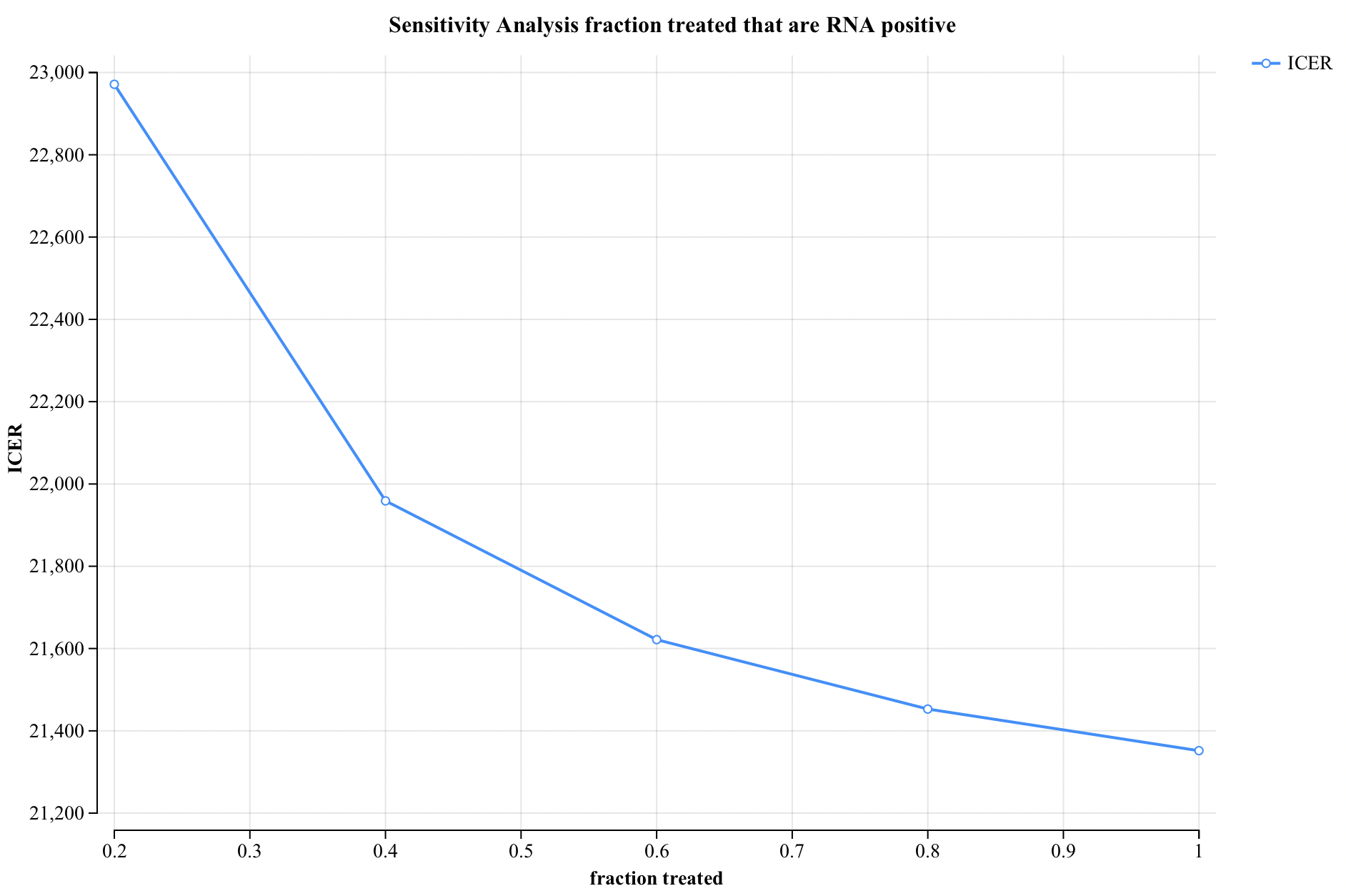


B)


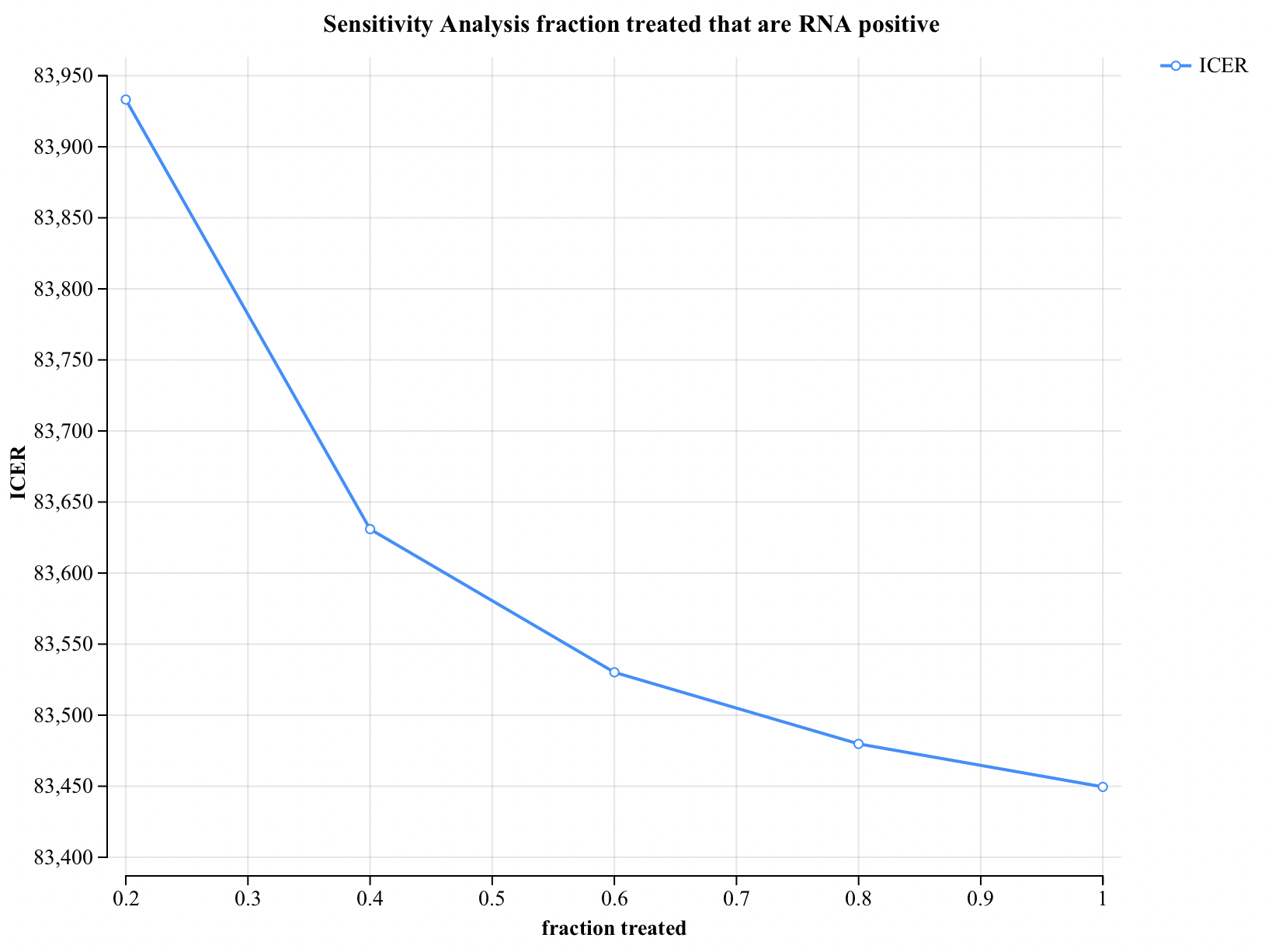


# Figure 9. Sensitivity analysis of hypothetical drug response rate


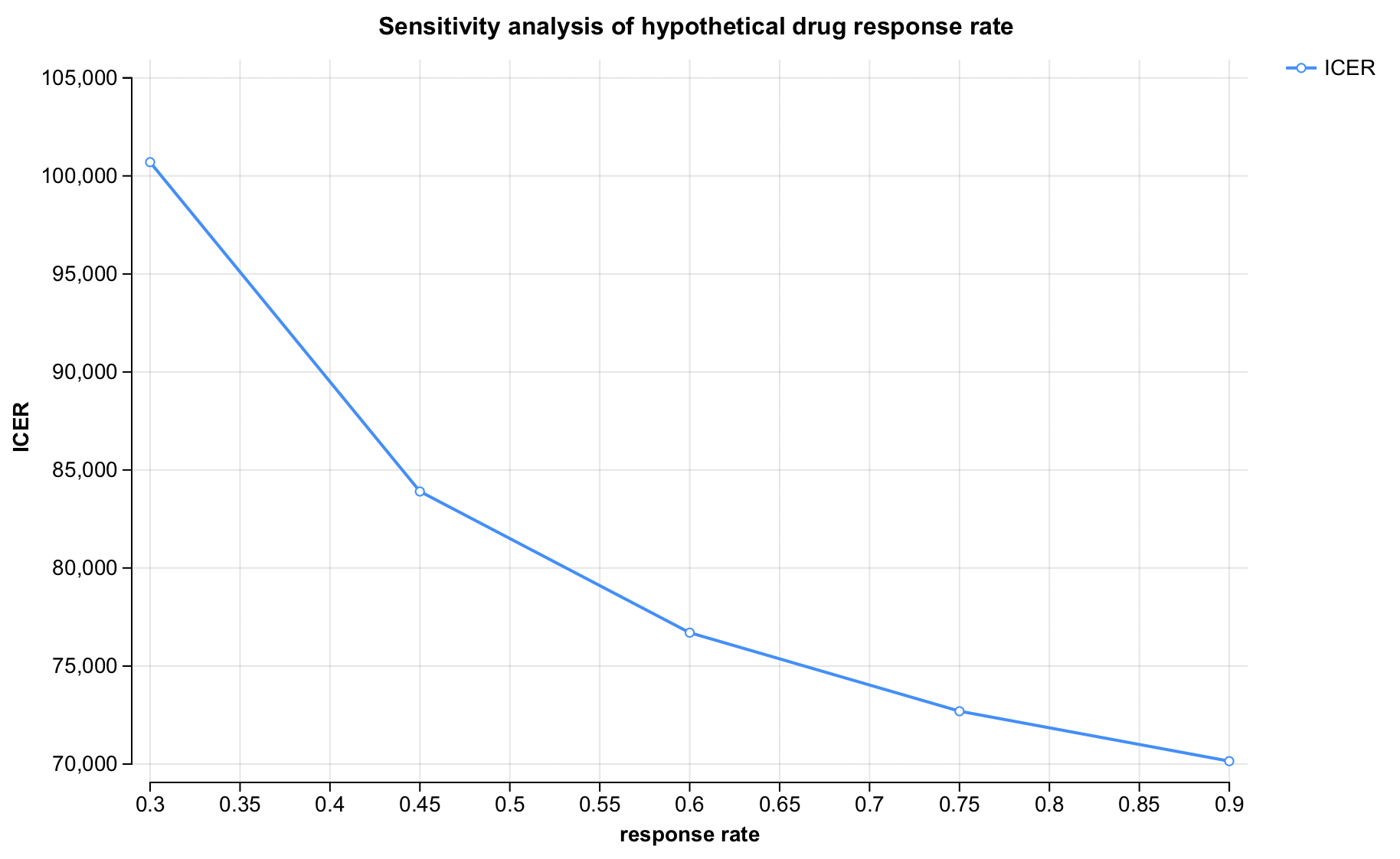


#

ICER, incremental cost-effectiveness ratio

# Figure 10. Cost-effectiveness acceptability curve


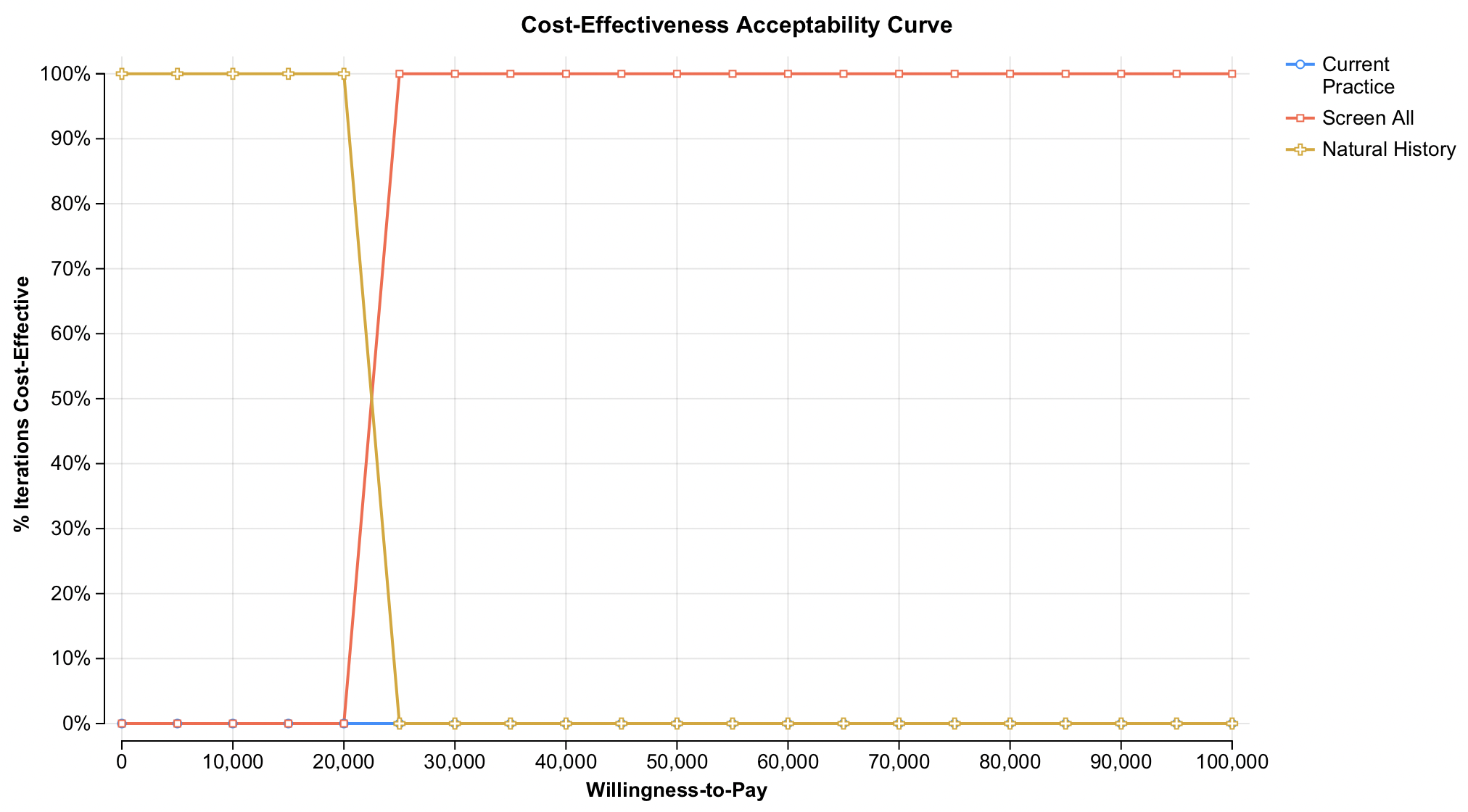


References

1. Kamal, H., et al., *Long-Term Study of Hepatitis Delta Virus Infection at Secondary Care Centers: The Impact of Viremia on Liver-Related Outcomes.* Hepatology, 2020. **72**(4): p. 1177-1190.

2. Alfaiate, D., et al., *Chronic hepatitis D and hepatocellular carcinoma: A systematic review and meta-analysis of observational studies.* J Hepatol, 2020. **73**(3): p. 533-539.

3. Lin, X., et al., *Chronic hepatitis B virus infection in the Asia-Pacific region and Africa: review of disease progression.* J Gastroenterol Hepatol, 2005. **20**(6): p. 833-43.

4. Wranke, A., et al., *Clinical long-term outcome of hepatitis D compared to hepatitis B monoinfection.* Hepatol Int, 2023.

5. Thiele, M., et al., *Large variations in risk of hepatocellular carcinoma and mortality in treatment naive hepatitis B patients: systematic review with meta-analyses.* PLoS One, 2014. **9**(9): p. e107177.

6. Organ Procurement and Transplantation Network. [cited 2023 Oct]; Available from: <https://optn.transplant.hrsa.gov/data/view-data-reports/build-advanced/>.

7. Ding, J. and Z. Wen, *Survival improvement and prognosis for hepatocellular carcinoma: analysis of the SEER database.* BMC Cancer, 2021. **21**(1): p. 1157.

8. Burra, P., et al., *Liver transplantation for HBV-related cirrhosis in Europe: an ELTR study on evolution and outcomes.* J Hepatol, 2013. **58**(2): p. 287-96.

9. Nguyen, M.H., et al., *Reduced Incidence of Hepatocellular Carcinoma in Cirrhotic and Noncirrhotic Patients With Chronic Hepatitis B Treated With Tenofovir-A Propensity Score-Matched Study.* J Infect Dis, 2019. **219**(1): p. 10-18.

10. Papatheodoridis, G.V., et al., *Risk of hepatocellular carcinoma in chronic hepatitis B: assessment and modification with current antiviral therapy.* J Hepatol, 2015. **62**(4): p. 956-67.

11. Zollner, C., et al., *Real-life experiences with bulevirtide for the treatment of hepatitis delta-48 weeks data from a German centre.* Liver Int, 2022. **42**(11): p. 2403-2407.
